# TAPESTRY study: impact of prior transurethral resection of the prostate on outcomes after robot-assisted radical prostatectomy: a propensity score-matched cohort study

**DOI:** 10.1007/s11701-026-03986-2

**Published:** 2026-09-19

**Authors:** Sebastian Lenart, Julia Zach, Georg Gutjahr, Lukas Oberhammer, Stephan Madersbacher, Anton Ponholzer, Clemens Mikulits, Andreas Banner

**Affiliations:** 1 Department of Urology and Andrology, St. John of God Hospital Vienna, Johannes-von-Gott Platz 1, Vienna, 1020 Austria; 2 Department of Health Sciences Research, Amrita Institute of Medical Science and Research Centre, Amrita Vishwa Vidyapeetham, Kochi, Kerala India; 3 Department of Urology and Andrology, Paracelsus Medical University, Salzburg, Austria; 4 Department of Urology and Andrology, Clinic Favoriten, Vienna, Austria

**Keywords:** Robot-assisted radical prostatectomy, Transurethral resection of the prostate, TURP, Prostate cancer, Propensity score matching, Surgical margins

## Abstract

Prior transurethral resection of the prostate (TURP) may distort bladder neck and apical anatomy and complicate robot-assisted radical prostatectomy (RARP). Its effect on anastomotic recovery and oncological control remains controversial. To evaluate perioperative, anastomotic, pathological, and available PSA outcomes after RARP in patients with and without prior TURP. We retrospectively analyzed a prospectively maintained database of 3,332 consecutive RARP procedures performed between August 2011 and December 2024. Ninety-seven patients with prior TURP were matched 1:3 to 291 TURP-naive controls using propensity scores derived from age, body mass index, clinical stage, ISUP grade, prostate-specific antigen (PSA), positive biopsy cores, and prostate volume. Comparative outcome analyses used available cases within the matched cohort. Baseline characteristics were well balanced after matching. Operative duration was similar between groups (median 135 vs. 139 min; *p* = .584). Prior TURP was associated with longer catheterization (7 [IQR 4.5–11.5] vs. 6 [4–7] days; *p* = .004), more cystographies (*p* = .004), more frequent catheterization beyond 7 days (33.7% vs. 20.1%; *p* = .011), and a higher rate of complications within 3 months (13.4% vs. 6.2%; *p* = .030). Cystographic leakage was numerically more frequent after TURP (25.4% vs. 14.8%; *p* = .061). Pathological stage, nodal positivity, positive surgical margin rate and length, and available PSA outcomes at 1 and 2 years were comparable. After Bonferroni adjustment, catheterization duration and the number of cystographies remained statistically significant. Prior TURP was associated primarily with greater postoperative anastomotic management reflected by longer catheterization and more cystographic follow-up; the observed increase in 3-month complications did not remain significant after correction for multiple comparisons. Available PSA data did not indicate a clear between-group difference, although incomplete follow-up precluded robust conclusions regarding biochemical control.

## Introduction

Radical prostatectomy remains a cornerstone in the treatment of localized and locally advanced prostate cancer, and robot-assisted radical prostatectomy (RARP) has become the predominant surgical approach in many high-income countries [1]. Improved visualization and instrument control have enabled refinements in dissection and reconstruction, including Retzius-sparing techniques [2, 3].

Due to demographic ageing and the overlapping epidemiology of benign prostatic hyperplasia (BPH) and prostate cancer (PC), a history of transurethral resection of the prostate (TURP) is encountered in a clinically relevant subset of men subsequently undergoing radical prostatectomy. TURP may cause fibrosis, scarring, loss of anatomical landmarks, and distortion of the bladder neck and prostatic apex. These changes can complicate bladder neck dissection, nerve-sparing, apical dissection, and vesicourethral anastomosis and may delay postoperative urinary recovery [4–6].

The available evidence is heterogeneous. Earlier open, laparoscopic, and mixed-modality studies suggested worse perioperative, pathological, and functional outcomes after prior TURP [4]. More recent RARP-specific series and meta-analyses still demonstrate increased technical and reconstructive demands, but report inconsistent effects on surgical margins, biochemical control, and longer-term continence [5–12].

High-quality comparative evidence remains limited because many studies include small post-TURP cohorts, heterogeneous surgical approaches, or incomplete adjustment for tumor stage, grade, PSA, biopsy burden, and prostate volume. Furthermore, detailed postoperative anastomotic and functional outcomes are often incompletely reported.

We therefore aimed to compare perioperative and anastomotic recovery, pathological findings, and available PSA outcomes in a contemporary 1:3 propensity score-matched RARP cohort of patients with and without prior TURP.

## Materials and methods

### Study design and population

This single-center retrospective cohort study was conducted at a high-volume tertiary urology center in Vienna, Austria. We analyzed a prospectively maintained institutional database initiated in August 2011. Between August 2011 and December 2024, 3,332 consecutive patients underwent RARP, of whom 97 patients (2.9%) with a documented history of TURP were compared with 3,235 TURP-naive patients. Prior TURP was identified from structured clinical documentation and preoperative medical history, irrespective of whether the procedure had been performed at our institution or externally.

All patients underwent RARP with or without pelvic lymph node dissection according to contemporary clinical indications and surgeon preference.

### Data collection and outcomes

Preoperative variables included age, body mass index (BMI), PSA, clinical tumor stage, ISUP grade group, number of positive biopsy cores, and prostate volume. Perioperative and pathological variables included operative duration, hospital stay, catheterization duration, cystographic leakage, number of cystographies, urinary retention after catheter removal, secondary anastomotic dehiscence, anastomotic stricture, postoperative complications, pathological tumor stage, nodal status, positive surgical margin status, and margin length. Cystographic assessment was performed between postoperative days 4 and 7 according to the surgeon’s preference. Retrograde cystography was performed under fluoroscopy by instillation of 100 mL of 50% Cystografin through the transurethral catheter. In the absence of anastomotic leakage, the catheter was removed immediately after cystography. If leakage was detected, the catheter was left in place until a subsequent cystogram confirmed resolution of the leakage. Repeat cystography was generally performed after 3–7 days, depending on the extent of leakage and the clinical course. Oncological follow-up included PSA values at 1 and 2 years after RARP among patients with available data.

The primary comparative outcomes were perioperative and anastomotic recovery, complications within 3 months, pathological findings, and PSA outcomes. Outcome-specific denominators were reported because completeness varied substantially across variables and follow-up time points.

### Functional outcomes

Functional outcomes were not included in the comparative analysis because standardized longitudinal continence and potency data were unavailable for the matched control group and follow-up in the prior-TURP group was incomplete and non-uniform.

### Statistical analysis

Missing values in the baseline covariates included in the propensity score model were handled using multiple imputation by chained equations, resulting in 1000 completed datasets. Propensity scores were subsequently estimated separately within each imputed dataset. Propensity scores were based on age, BMI, clinical stage, ISUP grade, PSA, number of positive biopsy cores, and prostate volume using MatchIt version 4.7.1 [13]. Covariate balance was assessed using standardized mean differences and a love plot [14]. Patients were matched in a 1:3 ratio using optimal variable-ratio matching implemented in optmatch version 0.10.8 [15]. Baseline estimates were pooled across imputed datasets using Rubin’s rules [16, 17]. Comparative outcome analyses were performed separately within each of the 1,000 propensity score-matched imputed datasets using available outcome data, and the resulting estimates were subsequently pooled across datasets using Rubin’s rules. No single representative matched dataset was used for hypothesis testing. Continuous variables are reported as median and interquartile range and were compared using the Mann–Whitney U test; categorical variables are reported as n/N (%) and were compared using Fisher’s exact or chi-square tests, as appropriate. No outcome imputation was performed. All tests were two-sided with *p* < .05 considered significant. Analyses were performed in R version 4.4.2. To account for multiple comparisons among the perioperative and anastomotic outcomes, Bonferroni-adjusted p-values were additionally calculated as a sensitivity analysis. Unadjusted p-values are reported for the primary analyses.

### Ethical approval

The study was approved by the institutional ethics committee of the Brothers of Mercy, Vienna, Austria (05/22), in September 2022 and was conducted in accordance with the Declaration of Helsinki. The requirement for written informed consent was waived for the retrospective analysis. Reporting followed the STROBE statement for cohort studies.

## Results

Across the 1,000 imputed datasets, 97 patients with prior TURP were matched to 291 TURP-naive controls in a 1:3 ratio.

### Baseline characteristics

Baseline characteristics were well balanced after matching. Mean age was 69.3 years in the TURP group and 69.1 years in controls; mean BMI was 27.4 kg/m^2^ in both groups. Clinical stage, ISUP grade, PSA, number of positive biopsy cores, and prostate volume were also comparable, and all propensity score covariates achieved standardized mean differences below 0.1 (Table 1). In addition, none of the matching covariates showed a statistically significant between-group difference after matching (all unadjusted *p* > .35).

**Table 1:** Baseline characteristics of patients with and without prior transurethral resection of the prostate before and after propensity score matching. Characteristics of the control and the TURP group are compared both unmatched (with the full control cohort of 3235 patients and all available cases of the variables) and matched (using 1000 completed, matched datasets of 291 control patients, where the results are pooled using Rubin’s rule)

	Control			TURP		TURP vs Control	
	Missing (original cohort)	Original (n = 3.235)	Matched (n = 291)	Missing (original cohort)	Matched (n = 97)	Unmatched (p-value)	Matched(p-value)
Age	0.0%	64.6 (7.1)	69.1 (6.2)	0.0%	69.3 (5.9)	< .001	.812
BMI	0.8%	27.3 (3.8)	27.2 (3.7)	0.0%	27.4 (3.7)	.767	.699
cT	7.0%			15.5%		.052	> 0.9
0		0.2%	0.0%		0.0%		
1b		0.0%	0.0%		7.3%		
1c		61.1%	72.0%		64.6%		
2a		17.6%	10.6%		14.6%		
2b		10.4%	7.3%		6.1%		
2c		9.6%	7.7%		4.9%		
3a		0.9%	2.0%		2.4%		
3b		0.2%	0.4%		0.0%		
ISUP	3.1%	2.2 (1.2)	2.2 (1.2)	1.0%	2.2 (1.1)	> 0.9	> 0.9
1		32.1%	28.0		27.1		
2		35.5%	41.9		43.8		
3		15.2%	14.0		13.5		
4		11.9%	9.7		9.4		
5		5.2%	6.5		6.2		
PSA	3.3%	9.3 (12.1)	8.8 (7.6)	4.1%	8.8 (11.3)	.706	>.9
Positive cores	9.5%	5.2 (3.4)	5.3 (3.4)	20.6%	4.9 (3.2)	.562	.353
Prostate volume	10.9%	41.7 (20.5)	40.1 (18.0)	17.5%	40.3 (27.7)	.647	> 0.9

### Perioperative and anastomotic outcomes

Median operative duration did not differ between the TURP and control groups (135 [IQR 100–178] vs. 139 [115–179.5] minutes; *p* = .584). Hospital stay was also comparable (7 [6–8] vs. 7 [6–7] days; *p* = .059). Immediate postoperative complications occurred in 8.2% and 3.8%, respectively (*p* = .100).

Anastomotic recovery required more postoperative management after prior TURP. Median catheterization duration was 7 days [IQR 4.5–11.5] after TURP versus 6 days [IQR 4–7] in controls (*p* = .004), and catheterization beyond 7 days occurred in 32/95 (33.7%) versus 57/283 (20.1%) patients (*p* = .011). The number of cystographies was also higher after TURP (median 1 [IQR 1–2] vs. 1 [1–1]; *p* = .004). Cystographic leakage or a non-watertight anastomosis was observed in 17/67 (25.4%) TURP patients and 26/176 (14.8%) controls (*p* = .061). Routine cystography was performed on postoperative days 4–7, with catheter removal immediately thereafter if no anastomotic leakage was detected; longer durations reflected leakage, repeat imaging, urinary retention, or other postoperative concerns. After Bonferroni adjustment for the 11 perioperative and anastomotic comparisons, the number of cystographies and catheterisation duration remained statistically significant (adjusted *p* = .044 for both), whereas the other associations did not.

Any complication within 3 months was more frequent after prior TURP (13/97 [13.4%] vs. 18/291 [6.2%]; *p* = .030). In the TURP group, the recorded events were urinary tract infection (5/97 [5.2%]), urinary retention after catheter removal (4/97 [4.1%]), prolonged catheterisation (3/97 [3.1%]), secondary anastomotic dehiscence (3/97 [3.1%]), primary anastomotic leakage/paravasation (1/97 [1.0%]), febrile infection (1/97 [1.0%]), and lymphocele (1/97 [1.0%]). In controls, events comprised urinary tract infection (6/291 [2.1%]), urinary retention after catheter removal (6/291 [2.1%]), primary anastomotic leakage/paravasation (5/291 [1.7%]), prolonged catheterisation (4/291 [1.4%]), lymphocele (4/291 [1.4%]), febrile infection (2/291 [0.7%]), pain (2/291 [0.7%]), and secondary anastomotic dehiscence (1/291 [0.3%]). Because individual patients could experience more than one event, category counts exceed the number of affected patients. No rectovesical fistula, thromboembolic event, anastomotic stricture, or inguinal hernia was documented within 3 months in either group (Table 2).

**Table 2:** Continuous variables are presented as median (interquartile range), and categorical variables as n/N (%). Denominators vary according to data availability. Outcome analyses were performed separately within each of the 1000 imputed and propensity score-matched datasets using available outcome data, and the resulting estimates were pooled across datasets using Rubin’s rules. No outcome imputation was performed. Unadjusted p-values are shown. Bonferroni adjustment for the 11 perioperative and anastomotic comparisons was performed as a sensitivity analysis; the number of cystographies and catheterisation duration remained statistically significant after adjustment (adjusted p = .044 for both). PSA ≥ .1 ng/mL represents a detectable postoperative PSA threshold and should not be interpreted as confirmed biochemical recurrence

Outcome	Control	TURP	Control, n	TURP, n	p-value
*Perioperative and anastomotic outcomes*					
Hospital stay in days, median (IQR)	7 (6–7)	7 (6–8)	261	90	.059
Operative duration, min, median (IQR)	139 (115–179.5)	135 (100–178)	271	91	.584
Immediate postoperative complication	11/291 (3.8%)	8/97 (8.2%)	291	97	.100
Any complication within 3 months	18/291 (6.2%)	13/97 (13.4%)	291	97	.030
Cystographic leakage/non-watertight anastomosis	26/176 (14.8%)	17/67 (25.4%)	176	67	.061
Number of cystographies, median (IQR)	1 (1–1)	1 (1–2)	189	69	.004
Catheterisation duration, days, median (IQR)	6 (4–7)	7 (4.5–11.5)	283	95	.004
Catheterisation > 7 days	57/283 (20.1%)	32/95 (33.7%)	283	95	.011
Urinary retention after catheter removal	6/291 (2.1%)	4/97 (4.1%)	291	97	.276
Secondary anastomotic dehiscence	1/291 (0.3%)	3/97 (3.1%)	291	97	.050
Anastomotic stricture within 3 months	0/291 (0.0%)	0/97 (0.0%)	291	97	1.000
*Pathological outcomes*					
Pathological stage distribution			285	97	.274
≤ pT2	194/285 (68.1%)	68/97 (70.1%)	285	97	.800
≥ pT3	91/285 (31.9%)	29/97 (29.9%)	285	97	.800
pN-positive among pN0/pN1	17/158 (10.8%)	6/53 (11.3%)	158	53	1.000
Positive surgical margin (R1)	58/286 (20.3%)	25/97 (25.8%)	286	97	.257
R1 length, mm, median (IQR)	3 (2–5.5)	4 (2–6)	56	24	.550
R1 in pT2 disease	20/194 (10.3%)	10/68 (14.7%)	194	68	.376
R1 in pT3 disease	38/91 (41.8%)	14/28 (50.0%)	91	28	.516
*PSA outcomes*					
PSA ≥ .1 ng/mL at 1 year	15/62 (24.2%)	7/30 (23.3%)	62	30	1.000
PSA ≥ .1 ng/mL at 2 years	13/47 (27.7%)	5/14 (35.7%)	47	14	.739

PSA outcomes were analysed among patients with available measurements at the respective follow-up time point. PSA ≥ .1 ng/mL represents a detectable postoperative PSA threshold and should not be interpreted as confirmed biochemical recurrence. No outcome imputation was performed

### Pathological and oncological outcomes

Pathological stage distribution and pN-positive disease were comparable between groups. Overall, positive surgical margins occurred in 25/97 patients (25.8%) with prior TURP and 58/286 controls (20.3%; *p* = .257). In analyses stratified by pathological stage, R1 margins were observed in 10/68 pT2 patients (14.7%) with prior TURP versus 20/194 controls (10.3%; *p* = .376), and in 14/28 pT3 patients (50.0%) with prior TURP versus 38/91 controls (41.8%; *p* = .516). The remaining R1 case in the TURP group occurred in a patient with pT4 disease. Median margin length among patients with R1 disease was 4 mm (IQR 2–6) after prior TURP and 3 mm (IQR 2–5.5) in controls (*p* = .550). 

Among patients with available PSA measurements, no statistically significant between-group differences in the proportion with PSA ≥ 0.1 ng/mL were observed at 1 or 2 years. However, follow-up completeness was limited, precluding robust conclusions regarding biochemical control. At 1 year, PSA was ≥ 0.1 ng/mL in 7/30 patients (23.3%) with prior TURP and 15/62 controls (24.2%; *p* = 1.000). At 2 years, the corresponding rates were 5/14 (35.7%) and 13/47 (27.7%; *p* = .739). Interpretation is limited by substantial follow-up missingness (Tables 2, 3).

**Table 3:** Key comparative evidence on RARP after TURP

Study	Study design	n	Operative/results	Complications	Oncologic outcomes	Functional outcomes
Hampton 2008	Retrospective matched RARP; TURP vs control	51/102	NR	NR	PSM 35.3% vs 17.7%; bladder-neck PSM 13.7% vs 2.0%	NR
Zugor 2012	Matched RARP; TURP vs control	80/80	Longer surgery and greater anastomotic burden	Clavien–Dindo ≥ 3: 10.0% vs 1.4%	Numerically less favourable	Delayed continence recovery
Su 2015	retrospective RARP; TURP vs control	49/2644	Comparable operative outcomes	No relevant difference	PSM 30.6% vs 21.0% (p =.110); BCR comparable	Continence comparable
Chaloupka 2021	Propensity matched; prior TURP vs control	172/513	NR	NR	PSM 34.5% vs 31.6%; BCR-free survival comparable	Continence 52.1% vs 71.2%; poorer HRQOL after prior TURP
Garg 2022	Matched RARP; TURP vs control	38/76	Longer surgery; more blood loss	Greater perioperative burden	PSM and BCR comparable	Worse continence at 3, 6, and 12 months
Bajpai 2022	Propensity matched RARP; TURP vs control	150/450	Greater technical complexity	No consistent difference	Oncologic outcomes comparable	Long-term function broadly comparable
Tappero 2023	RS RARP; TURP vs control	99/1287	Operative time 202 vs 190 min	13% vs 13%	PSM 23% vs 17% (p=.10)	Continence 40% vs 67% immediately and 68% vs 94% at 12 months
Jaber 2024	Retrospective RARP; TURP vs control	231/677	Comparable operative outcomes	Comparable	PSM and BCR comparable	Continence and potency comparable
Carbin 2024	Matched RARP; TURP vs control	30/90	Longer console time trend; less complete nerve sparing	No significant overall difference	PSM and BCR comparable	Continence comparable
Hu 2025	Updated RARP meta-analysis	15 studies; 6748	Operative time +26.6 min; bladder-neck reconstruction OR 8.38	Major complications OR 1.94	PSM OR 1.25; 1-year BCR comparable	Less favourable urinary recovery.
Current study (TAPESTRY)	Propensity matched RARP; TURP vs control	97/291	Operative time 135 vs 139 min; catheterisation 7 vs 6 days; > 7 days 33.7% vs 20.1%	3-month complications 13.4% vs 6.2%	PSM 25.8% vs 20.3%; PSA outcomes comparable	Not reliably assessable because standardized comparative data were unavailable

BCR, biochemical recurrence; BPE, benign prostatic enlargement; HRQOL, health-related quality of life; NR, not reported; PSM, positive surgical margin; RARP, robot-assisted radical prostatectomy; TURP, transurethral resection of the prostate. Meta-analyses include overlapping primary studies

## Discussion

In this propensity score-matched cohort, prior TURP was not associated with longer operative duration or adverse pathological findings, but it was associated with a greater postoperative anastomotic management burden. Patients with prior TURP had longer catheterization, underwent more cystographic examinations, more frequently required catheterization beyond 7 days, and experienced more complications within 3 months. Cystographic leakage and secondary anastomotic dehiscence were also numerically more frequent, although the number of events was limited. Available PSA outcomes at 1 and 2 years were comparable. After adjustment for multiple comparisons, only catheterisation duration and the number of cystographies remained statistically significant, whereas the other associations should be interpreted as exploratory.

These results refine the concept of technical complexity after TURP. A broad meta-analysis by Li et al. included 15 retrospective studies and 6,840 patients across surgical approaches and reported worse perioperative and functional outcomes after prior TURP [4]. RARP-specific meta-analyses by Gu et al. and Liu et al. similarly demonstrated longer operative and catheterization times and less favorable functional recovery in post-TURP patients, whereas oncological endpoints were less consistently affected. Gu et al. included 10 studies with 683 post-TURP patients and 4,039 controls and reported longer operative and catheterization times after TURP [5] whereas Liu et al. included 8 comparative trials with a total of 4186 participants and found longer surgery time, catheterization times and higher bladder neck reconstruction rates after TURP [9]. A recent systematic review and meta-analysis by Hu et al. confirmed that RARP after prior TURP was associated with longer operative time, greater blood loss, and a higher likelihood of bladder neck reconstruction. The authors also reported less favourable urinary recovery, whereas biochemical recurrence outcomes were comparable with those of TURP-naive patients [18]. In our matched cohort, operative time was similar, whereas the difference was concentrated in catheterization and repeat cystographic assessment. This finding suggests that prior TURP may affect reconstructive healing and postoperative management more consistently than the duration of resection itself.

Individual robotic series also illustrate the heterogeneity of this population. Gupta et al. studied 158 patients, including 26 with prior TURP, and reported greater technical difficulty during RARP in men with previous TURP [19]. Hung et al. compared 16 post-TURP patients with 184 controls and found more bladder neck reconstruction and more major complications, although 12-month continence remained high in both groups [11]. Garg et al. likewise reported less favourable perioperative and continence outcomes after prior TURP in a matched RARP cohort. Other contemporary matched series, however, have found broadly comparable oncological and long-term functional outcomes, despite evidence of greater operative complexity or reduced feasibility of nerve-sparing surgery [20, 21]. In contrast, Jaber et al. found no significant differences in perioperative safety, continence, potency, positive surgical margins, or biochemical recurrence, suggesting that experienced teams may achieve comparable outcomes despite altered anatomy [22]. Beyond surgical approach, previous studies have also reported impaired health-related quality of life and identified patient- and treatment-related factors as potential determinants of functional recovery after radical prostatectomy in men with prior transurethral prostate surgery [23, 24].

The choice of surgical approach may also influence functional recovery in patients with previous TURP. In a contemporary cohort of 65 post-TURP patients, Schütz et al. found that Retzius-sparing RARP (RS RARP) improved immediate continence compared with an anterior approach, whereas longer-term continence and 24-month biochemical recurrence-free survival were comparable [7]. In contrast, Tappero et al., who compared patients undergoing RS RARP after TURP with TURP-naiive controls, reported lower immediate continence recovery (40% vs. 67%) and lower 12-month continence recovery (68% vs. 94%) compared with TURP-naive patients [25]. These findings indicate that RS RARP may improve recovery relative to an anterior approach within the post-TURP population, but it may not completely eliminate the functional disadvantage associated with previous TURP. In the context of predominantly small, uncontrolled post-TURP series, our study adds evidence from one of the larger single-centre cohorts and strengthens comparative interpretation through the inclusion of an internally matched control group.

The pathological and PSA findings in our study were reassuring. Neither pathological stage, nodal positivity, positive surgical margin rate, nor positive margin length differed significantly between the groups. Among patients with available follow-up, the proportion with PSA ≥ 0.1 ng/mL did not differ significantly between groups at 1 or 2 years. However, the limited number of evaluable patients precludes robust conclusions regarding biochemical recurrence-free survival. These observations are consistent with contemporary robotic reports suggesting that prior TURP does not necessarily compromise cancer control when baseline disease characteristics are balanced [5, 6, 8, 9, 21]. However, the small number of patients with PSA data beyond 1 year precludes robust conclusions regarding biochemical recurrence-free survival.

From a clinical perspective, a history of TURP should prompt careful counselling regarding the potential need for prolonged catheterization and repeated cystographic assessment. At our center, the standard catheterization period was 4–7 days, provided that cystography showed no evidence of anastomotic leakage. The higher median catheterization time and wider interquartile range in the TURP group primarily reflected leakage, repeated imaging, urinary retention, or other postoperative concerns. The absence of significant differences in operative time or pathological outcomes indicates that the additional burden associated with prior TURP predominantly emerged during anastomotic recovery rather than during oncological resection. This interpretation is consistent with meta-analytic evidence suggesting that the principal effects of previous TURP are technical and functional rather than clearly oncological.

This study has several limitations. Its retrospective, single-centre design introduces the possibility of residual confounding and limits external generalisability. The extent of the previous TURP and the interval between TURP and RARP were incompletely documented, and surgeon-specific effects could not be fully excluded. All outcome analyses were based on complete cases, and PSA and functional follow-up were substantially incomplete. Standardized comparative longitudinal data on continence and potency were unavailable; therefore, functional outcomes could not be reliably assessed.

Nevertheless, the study includes one of the larger post-TURP RARP cohorts reported to date, uses a rigorously matched 1:3 control group, and provides a detailed assessment of catheterisation, cystographic examinations, anastomotic events, pathological outcomes, and intermediate-term PSA findings.

## Conclusion

In this propensity score-matched cohort, prior TURP was associated with longer catheterisation, more cystographic follow-up, and more complications within 3 months, but not with longer operative duration, adverse pathological findings, or different available PSA outcomes at 1 and 2 years. Prior TURP should therefore be regarded primarily as a marker of increased postoperative anastomotic management rather than inferior oncological suitability for RARP.

## Data Availability

The datasets generated and/or analyzed during the current study are not publicly available due to patient privacy and institutional data protection regulations, but are available from the corresponding author upon reasonable request and subject to approval by the responsible institutional authorities.
